# Supplementary material for: Plasma Metabolomic Profiling in 1391 Subjects with Overweight and Obesity from the SPHERE Study
Source: Metabolites. 2021 Mar 24;11(4):194. doi: 10.3390/metabo11040194 (PMC8064361; doi:10.3390/metabo11040194)
Supplement: Supplementary file 1 [file metabolites-11-00194-s001.zip › Table_S14.pdf]

Supplementary material

Table S14 – Summary information about findings of our study, including the comparison with reference values reported by Trabado et al. 2017 (Trabado et al., 2017), the percentage of variation identified in the Tobit models, and a comparison with other studies.

**Chemical characteristic**	**Differences with Trabado et al. 2017**	**Tobit (Δ%)**	**Studies which found similar associations**	**Studies with findings in disagreements with ours**
**Amino acids positively associated with BMI**				
Valine (BCAA)	our results> reference values	+23.2	(Bagheri et al., 2019, 2018; Cirulli et al., 2019; Dunn et al., 2015; Ho et al., 2016; Kim et al., 2010; Libert et al., 2018; Newgard et al., 2009; Wang et al., 2018; Xie et al., 2014)	
Isoleucine (BCAA)		+20.0		
Leucine (BCAA)	our results> reference values	+15.0		
Tyrosine (aromatic aa)	our results>> reference values	+29.8	(Cirulli et al., 2019; Dunn et al., 2015; Ho et al., 2016; Libert et al., 2018; Rangel-Huerta et al., 2019; Wang et al., 2018)	
Phenylalanine (aromatic aa)	our results>> reference values	+20.4		
Tryptophan (aromatic aa)				It was higher in (Kim et al., 2010; Wang et al., 2018; Yu et al., 2018)
Alanine (hydrophobic aa)	our results> reference values	+18.9	(Bagheri et al., 2019; Dunn et al., 2015; Ho et al., 2016; Libert et al., 2018)	
Proline (hydrophobic aa)		+18.1	(Bagheri et al., 2019; Dunn et al., 2015)	
glutamic acid (negative polar aa)	our results>> reference values	+17.1	(Bagheri et al., 2019, 2018; Carayol et al., 2017; Wang et al., 2018; Zhao et al., 2016)	
Lysine (positive polar aa)	our results> reference values	+7.1	(Dunn et al., 2015; Libert et al., 2018)	
Ornithine (positive polar aa)	our results>> reference values	+9.2	(Dunn et al., 2015)	
**Amino acids negatively associated with BMI**				

Asparagine (uncharged polar aa)	reference values>our results	-16.1	(Bagheri et al., 2019; Dunn et al., 2015)	
Glycine (uncharged polar aa)		-13.6		It was higer in (Oberbach et al., 2011)
Histidine (Charged polar aa)		-11.8		
Serine (Uncharged polar aa)		-10.0	(Bagheri et al., 2019; Dunn et al., 2015)	
Citidine (polar aa)		-6.2		It was not significative in (Dunn et al., 2015)
Threonine (Uncharged polar aa)				It was not significative in (Dunn et al., 2015)
Arginine (Positive polar aa)	reference values>>our results			
**Biogenic amines positively associated with BMI**				
Kynurenine	our results>> reference values	+16.1	(Carayol et al., 2017; Ho et al., 2016; Zhao et al., 2016)	
Aminoadipic acid		+18.5		
4-hydroxyproline		+8.0		
**Biogenic amines negatively associated with BMI**				
Serotonin		-7.4		
Creatinine	reference values>our results	-6.1		Higher in (Dunn et al., 2015)
N-acetylornithine		-9.2		
**Acylcarnitines positively associated with BMI**				
C0		+12.6	(Cirulli et al., 2019; Xie et al., 2014)	Lower in (Kim et al., 2010)
C3		+12.2	(Cirulli et al., 2019; Kim et al., 2010; Libert et al., 2018; Xie et al., 2014)	
C2		+9.1		

C5				
C18.1	our results> reference values	+5.7 (FDR P=0.070)		
**Acylcarnitines negatively associated with BMI**				
C12		24.5		
**LysoPC positively associated with BMI**				
lysoPC a C16:1		+7.3	(Bagheri et al., 2019)	
**LysoPC negatively associated with BMI**				
lysoPC a C26:0	reference values>>our results			
lysoPC a C28:0	reference values>>our results			
lysoPC a C18:2	reference values>our results	-23.3	(Bagheri et al., 2019, 2018; Carayol et al., 2017; Ho et al., 2016; Tulipani et al., 2016; Wang et al., 2018)	
lysoPC a C28:1	reference values>our results			
lysoPC a C18:1		-15.9	(Bagheri et al., 2019, 2018; Carayol et al., 2017; Ho et al., 2016; Kim et al., 2010; Tulipani et al., 2016; Wang et al., 2018)	
lysoPC a C17:0		-14.3	(Tulipani et al., 2016)	
**PC aa positively associated with BMI**				
PC aa C38:3	our results>reference values	+21.1	(Bagheri et al., 2019, 2018; Carayol et al., 2017; Ho et al., 2016)	
PC aa C40:4		+14.3		
PC aa C32:1		+12.4	(Bagheri et al., 2019, 2018; Carayol et al., 2017)	(Oberbach et al., 2011)
PC aa C38:4		+7.6	(Carayol et al., 2017)	
PC aa C40:5		+6.9		(Oberbach et al., 2011)
**PC aa negatively associated with BMI**				
PC aa C38:6	reference values>>our results	-12.9	(Ho et al., 2016)	
PC aa C38:0	reference values>>our results	-11.7		

PC aa C36:0	reference values>our results	-11.7		
PC aa C42:5	reference values>>our results	-11.6		
PC aa C36:6	reference values>>our results	-10.6		
PC aa C34:2	reference values>>>our results	-10.5		
PC aa C42:1	reference values>>>our results	-10.0	(Carayol et al., 2017)	
PC aa C42:6	reference values>>our results	-10.5		
PC aa C40:3	reference values>>our results	-8.8		
PC aa C42:2	reference values>>our results	-9.6	(Carayol et al., 2017)	
PC aa C42:0	reference values>>>our results	-8.4	(Carayol et al., 2017)	Higher in (Oberbach et al., 2011)
PC aa C40:2	reference values>>our results	-7.3		
PC aa C28:1				
**PC ae negatively associated with BMI**				
PC ae C36:2	reference values>>our results	-20.5		
PC ae C34:3	reference values>>our results	-20.2	(Bagheri et al., 2019)	
PC ae C34:2	reference values>>our results	-18.2		
PC ae C40:6	reference values>>our results	-16.1	(Bagheri et al., 2019)	
PC ae C40:1	reference values>>our results	-16.0		
PC ae C38:0	reference values>>our results	-15.2		

PC ae C36:3	reference values>>our results	-12.3		
PC ae C42:3	reference values>>our results	-11.9	(Carayol et al., 2017)	
PC ae C36:1	reference values>>our results	-10.6		
PC ae C30:0	reference values>>our results	-10.6		
PC ae C40:5	reference values>>our results	-10.5	(Carayol et al., 2017)	
PC ae C42:2	reference values>>our results	-10.1		
PC ae C44:6	reference values>>our results	-10.3	(Carayol et al., 2017)	
PC ae C34:1	reference values>>our results	-9.5		
PC ae C38:6	reference values>>our results	-9.4		
PC ae C32:1	reference values>>our results	-9.0		
PC ae C42:1	reference values>>our results	-8.9		
PC ae C38:5	reference values>>our results	-8.5		
PC ae C42:4	reference values>>our results	-8.4	(Carayol et al., 2017)	
PC ae C34:0	reference values>>our results	-7.8		
PC ae C36:5	reference values>>our results	-7.9		
PC ae C44:5	reference values>>our results	-7.8	(Carayol et al., 2017)	
PC ae C42:5	reference values>>our results	-7.2	(Carayol et al., 2017)	

PC ae C36:0	reference values>>our results	-7.1		
PC ae C32:2	reference values>>our results	-6.7		
PC ae C38:4	reference values>>our results	-6.9	(Bagheri et al., 2019)	
PC ae C38:2	reference values>>our results	-6.1	(Carayol et al., 2017)	
**SM positively associated with BMI**				
SM C18:1	our results> reference values	8.3	(Ho et al., 2016)	
SM C16:1		7.0	(Ho et al., 2016)	
SM C18:0	our results> reference values		(Carayol et al., 2017; Ho et al., 2016)	
**SM negatively associated with BMI**				
SM C24:1		-10.5		
SM C16:0		-9.7		
SM C26:1	reference values>>our results	-8.9		
SM (OH) C22:2		-8.3		
SM (OH) C16:1		-6.7		
SM (OH) C14:1		-5.9		
SM C26:0	reference values>>our results			

References

Bagheri, M., Djazayery, A., Farzadfar, F., Qi, L., Yekaninejad, M.S., Aslibekyan, S., Chamari, M., Hassani, H., Koletzko, B., Uhl, O., 2019. Plasma metabolomic profiling of amino acids and polar lipids in Iranian obese adults. Lipids Heal. Dis 18, 94. https://doi.org/10.1186/s12944-019-1037-0

Bagheri, M., Farzadfar, F., Qi, L., Yekaninejad, M.S., Chamari, M., Zeleznik, O.A., Kalantar, Z., Ebrahimi, Z., Sheidaie, A., Koletzko, B., Uhl, O., Djazayery, A., 2018. Obesity-Related Metabolomic Profiles and Discrimination of Metabolically Unhealthy Obesity. J Proteome Res 17, 1452–1462. https://doi.org/10.1021/acs.jproteome.7b00802

Carayol, M., Leitzmann, M.F., Ferrari, P., Zamora-Ros, R., Achaintre, D., Stepien, M., Schmidt, J.A., Travis, R.C., Overvad, K., Tjønneland, A., Hansen, L., Kaaks, R., Kühn, T., Boeing, H., Bachlechner, U., Trichopoulou, A., Bamia, C., Palli, D., Agnoli, C., Tumino, R., Vineis, P., Panico, S., Quirós, J.R., Sánchez-Cantalejo, E., Huerta, J.M., Ardanaz, E., Arriola, L., Agudo, A., Nilsson, J., Melander, O., Bueno-de-Mesquita, B., Peeters, P.H., Wareham, N., Khaw, K.T., Jenab, M., Key, T.J., Scalbert, A., Rinaldi, S., 2017. Blood Metabolic Signatures of Body Mass Index: A Targeted Metabolomics Study in the EPIC Cohort. J Proteome Res 16, 3137–3146. https://doi.org/10.1021/acs.jproteome.6b01062

Cirulli, E.T., Guo, L., Leon Swisher, C., Shah, N., Huang, L., Napier, L.A., Kirkness, E.F., Spector, T.D., Caskey, C.T., Thorens, B., Venter, J.C., Telenti, A., 2019. Profound Perturbation of the Metabolome in Obesity Is Associated with Health Risk. Cell Metab 29, 488-500.e2. https://doi.org/10.1016/j.cmet.2018.09.022

Dunn, W.B., Lin, W., Broadhurst, D., Begley, P., Brown, M., Zelena, E., Vaughan, A.A., Halsall, A., Harding, N., Knowles, J.D., Francis-McIntyre, S., Tseng, A., Ellis, D.I., O'Hagan, S., Aarons, G., Benjamin, B., Chew-Graham, S., Moseley, C., Potter, P., Winder, C.L., Potts, C., Thornton, P., McWhirter, C., Zubair, M., Pan, M., Burns, A., Cruickshank, J.K., Jayson, G.C., Purandare, N., Wu, F.C., Finn, J.D., Haselden, J.N., Nicholls, A.W., Wilson, I.D., Goodacre, R., Kell, D.B., 2015. Molecular phenotyping of a UK population: defining the human serum metabolome. Metabolomics 11, 9–26. https://doi.org/10.1007/s11306-014-0707-1

Ho, J.E., Larson, M.G., Ghorbani, A., Cheng, S., Chen, M.H., Keyes, M., Rhee, E.P., Clish, C.B., Vasan, R.S., Gerszten, R.E., Wang, T.J., 2016. Metabolomic Profiles of Body Mass Index in the Framingham Heart Study Reveal Distinct Cardiometabolic Phenotypes. PLoS One 11, e0148361. https://doi.org/10.1371/journal.pone.0148361

Kim, J.Y., Park, J.Y., Kim, O.Y., Ham, B.M., Kim, H.J., Kwon, D.Y., Jang, Y., Lee, J.H., 2010. Metabolic profiling of plasma in overweight/obese and lean men using ultra performance liquid chromatography and Q-TOF mass spectrometry (UPLC-Q-TOF MS). J Proteome Res 9, 4368–4375. https://doi.org/10.1021/pr100101p

Libert, D.M., Nowacki, A.S., Natowicz, M.R., 2018. Metabolomic analysis of obesity, metabolic syndrome, and type 2 diabetes: amino acid and acylcarnitine levels change along a spectrum of metabolic wellness. PeerJ 6, e5410. https://doi.org/10.7717/peerj.5410

Newgard, C.B., An, J., Bain, J.R., Muehlbauer, M.J., Stevens, R.D., Lien, L.F., Haqq, A.M., Shah, S.H., Arlotto, M., Slentz, C.A., Rochon, J., Gallup, D., Ilkayeva, O., Wenner, B.R., Yancy, W.S., Eisenson, H., Musante, G., Surwit, R.S., Millington, D.S., Butler, M.D., Svetkey, L.P., 2009. A branched-chain amino acid-related metabolic signature that differentiates obese and lean humans and contributes to insulin resistance. Cell Metab 9, 311–326.

https://doi.org/10.1016/j.cmet.2009.02.002

Oberbach, A., Blüher, M., Wirth, H., Till, H., Kovacs, P., Kullnick, Y., Schlichting, N., Tomm, J.M., Rolle-Kampczyk, U., Murugaiyan, J., Binder, H., Dietrich, A., von Bergen, M., 2011. Combined proteomic and metabolomic profiling of serum reveals association of the complement system with obesity and identifies novel markers of body fat mass changes. J Proteome Res 10, 4769–4788. https://doi.org/10.1021/pr2005555

Rangel-Huerta, O.D., Pastor-Villaescusa, B., Gil, A., 2019. Are we close to defining a metabolomic signature of human obesity? A systematic review of metabolomics studies. Metabolomics 15, 93. https://doi.org/10.1007/s11306-019-1553-y

Trabado, S., Al-Salameh, A., Croixmarie, V., Masson, P., Corruble, E., Fève, B., Colle, R., Ripoll, L., Walther, B., Boursier-Neyret, C., Werner, E., Becquemont, L., Chanson, P., 2017. The human plasma-metabolome: Reference values in 800 French healthy volunteers; impact of cholesterol, gender and age. PLoS One 12, e0173615. https://doi.org/10.1371/journal.pone.0173615

Tulipani, S., Palau-Rodriguez, M., Miñarro Alonso, A., Cardona, F., Marco-Ramell, A., Zonja, B., de Alda, M., Muñoz-Garach, A., Sanchez-Pla, A., Tinahones, F.J., Andres-Lacueva, C., 2016. Biomarkers of Morbid Obesity and Prediabetes by Metabolomic Profiling of Human Discordant Phenotypes. Clin Chim Acta 463, 53–61. https://doi.org/10.1016/j.cca.2016.10.005

Wang, S.M., Yang, R.Y., Wang, M., Ji, F.S., Li, H.X., Tang, Y.M., Chen, W.X., Dong, J., 2018. Identification of serum metabolites associated with obesity and traditional risk factors for metabolic disease in Chinese adults. Nutr Metab Cardiovasc Dis 28, 112–118. https://doi.org/10.1016/j.numecd.2017.09.009

Xie, G., Ma, X., Zhao, A., Wang, C., Zhang, Y., Nieman, D., Nicholson, J.K., Jia, W., Bao, Y., 2014. The metabolite profiles of the obese population are gender-dependent. J Proteome Res 13, 4062–4073. https://doi.org/10.1021/pr500434s

Yu, H.T., Fu, X.Y., Xu, B., Zuo, L.L., Ma, H.B., Wang, S.R., 2018. Untargeted metabolomics approach (UPLC-Q-TOF-MS) explores the biomarkers of serum and urine in overweight/obese young men. Asia Pac J Clin Nutr 27, 1067–1076. https://doi.org/10.6133/apjcn.052018.07

Zhao, Q., Zhu, Y., Best, L.G., Umans, J.G., Uppal, K., Tran, V.T., Jones, D.P., Lee, E.T., Howard, B. V, Zhao, J., 2016. Metabolic Profiles of Obesity in American Indians: The Strong Heart Family Study. PLoS One 11, e0159548. https://doi.org/10.1371/journal.pone.0159548
