# Supplementary material for: Plasma Metabolomic Profiling in 1391 Subjects with Overweight and Obesity from the SPHERE Study
Source: Metabolites. 2021 Mar 24;11(4):194. doi: 10.3390/metabo11040194 (PMC8064361; doi:10.3390/metabo11040194)
Supplement: Supplementary file 1 [file metabolites-11-00194-s001.zip › Table_S02-S07.pdf]

Supplementary material

Table S2 – Results obtained from the analyses of the 21 amino acids and the sugar. Results are reported as mean, standard deviation, 25th and 75th percentile (inter-quartile range), minimum and maximum (extreme values). For each compound the limit of detection is reported along with the number of subjects with quantifiable concentrations.

	**Mean±SD (µmol/L)**	**Median**	**Inter-quartile Range**	**Extreme values**	**LOD**	**N<LOD (%)**
L-Alanine	367.52±84.57	361.00	[308.00;418.00]	(167.00;755.00)	1.00	0 (0%)
L-Arginine	53.61±17.83	51.90	[41.20;64.33]	(8.57;128.00)	0.50	0 (0%)
L-Asparagine	42.74±8.66	42.00	[36.75;47.90]	(18.80;104.00)	0.46	0 (0%)
L-Aspartic acid	7.67±5.22	6.84	[4.90;9.42]	(<LOD;129.00)	0.91	42 (3%)
L-Citrulline	29.27±8.79	28.30	[23.60;33.90]	(<LOD;100.00)	0.40	1 (0.1%)
L-Glutamine	623.17±118.03	624.00	[554.00;701.00]	(61.90;1025.00)	0.53	0 (0%)
L-Glutamic acid	87.83±44.65	80.10	[58.80;107.00]	(13.90;628.00)	0.55	0 (0%)
Glycine	239.56±76.05	225.00	[190.00;271.00]	(74.60;748.00)	0.08	0 (0%)
L-Histidine	85.93±13.93	85.20	[76.40;94.55]	(26.90;182.00)	0.50	0 (0%)
L-Isoleucine	86.02±22.09	82.50	[71.18;96.93]	(32.80;210.00)	0.33	0 (0%)
L-Leucine	174.24±43.26	168.00	[146.00;195.00]	(68.20;603.00)	1.50	0 (0%)
L-Lysine	232.73±70.39	235.00	[184.00;279.00]	(68.50;470.00)	0.50	0 (0%)
L-Methionine	25.67±6.75	24.90	[21.88;28.60]	(10.10;135.00)	0.10	0 (0%)
L-Ornithine	110.68±33.97	107.00	[86.15;130.00]	(33.30;310.00)	0.50	0 (0%)
L-Phenylalanine	73.58±14.38	72.10	[63.40;81.20]	(40.90;169.00)	0.02	0 (0%)
L-Proline	205.63±74.60	191.00	[154.00;239.00]	(72.00;618.00	0.49	0 (0%)
L-Serine	114.58±27.07	112.00	[96.10;130.25]	(35.60;295.00)	0.79	0 (0%)
L-Threonine	128.64±33.25	124.00	[106.00;145.00]	(60.40;355.00)	0.40	0 (0%)
L-Tryptophan	66.51±14.15	65.45	[56.30;75.50]	(28.50;126.00)	0.06	0 (0%)
L-Tyrosine	80.35±22.39	78.25	[65.50;92.55]	(25.50;275.00)	0.01	0 (0%)

L-Valine	288.24±60.81	282.00	[246.00;326.25]	(133.00;548.00)	0.50	0 (0%)
hexose	4976.99±1301.73	4763.50	[4288.25;5239.00]	(2838.00;24381.00)	276.00	0 (0%)

SD = standard deviation, LOD = limit of detection, LLOQ = lower limit of quantitation

Table S3 – Results obtained from the analyses of the 21 biogenic amines. Results are reported as mean, standard deviation, 25th and 75th percentile (inter-quartile range), minimum and maximum (extreme values). For each compound the limit of detection is reported along with the number of subjects with quantifiable concentrations.

	**Mean±SD (µmol/L)**	**Median**	**Inter-quartile Range**	**Extreme values**	**LOD**	**N<LOD (%)**
N-Acetylornithine	0.80±1.26	0.55	[<LOD;1.05]	(<LOD;25.90)	0.05	441 (31.7%)
Asymmetric dimethylarginine	0.37±0.55	0.38	[<LOD;0.56]	(<LOD;15.50)	0.08	540 (38.8%)
Alpha-Aminoadipic acid	0.61±0.64	0.45	[<LOD;0.98]	(<LOD;5.89)	0.08	595 (42.7%)
cis-4-Hydroxy-L-proline	0.13±0.61	<LOD	<LOD	(<LOD;18.30)	0.10	1278 (91.7%)
Carnosine	0.03±0.04	<LOD	<LOD	(<LOD;0).98	0.02	1337 (96%)
Creatinine	60.46±21.39	58.05	[50.40;66.60]	(10.40;609.00)	0.002	0 (0%)
L-Dopa	0.22±0.73	<LOD	[<LOD;0.45]	(<LOD;24.50)	0.001	881 (63.2%)
Dopamine	0.12±0.34	<LOD	<LOD	(<LOD;12.40)	0.10	1243 (89.2%)
Histamine	0.09±0.21	<LOD	<LOD	(<LOD;0.91)	0.01	1167 (83.8%)
L-Kynurenine	2.94±1.15	2.82	[2.35;3.37]	(0.23;31.90)	0.01	0 (0%)
Methionine sulfoxide	0.60±0.88	<LOD	[<LOD;0.76]	(<LOD;26.80)	0.30	867 (62.2%)
3-Nitrotyrosine	<LOD	<LOD	<LOD	(<LOD;0.37)	0.06	1378 (98.9%)
1-Phenylethylamine	0.03±0.20	<LOD	<LOD	(<LOD;7.37)	0.02	1389 (99.7%)
Putrescine	0.08±0.18	0.06	[<LOD;0.11]	(<LOD;6.01)	0.001	484 (34.7%)
Symmetric dimethylarginine	0.37±2.03	0.29	[<LOD;0.53]	(<LOD;74.50)	0.01	646 (46.4%)
Serotonin	0.43±0.56	0.33	[0.20;0.53]	(<LOD;14.70)	0.01	66 (4.7%)
Spermidine	0.25±0.20	0.23	[<LOD;0.35]	(<LOD;4.63)	0.08	468 (33.6%)
Spermine	0.11±0.10	<LOD	<LOD	(<LOD;1.79)	0.08	1125 (80.8%)
4-Hydroxyproline	10.09±6.15	8.39	[6.40;11.90]	(2.10;70.00)	0.07	0 (0%)
Taurine	106.56±51.33	94.60	[74.60;123.00]	(30.30;472.00)	0.06	0 (0%)
Total dimethylarginine	0.99±0.49	1.01	[0.85;1.15]	(<LOD;10.30)	0.01	101 (7.3%)

SD = standard deviation, LOD = limit of detection, LLOQ = lower limit of quantitation

Table S4 - Results obtained from the analyses of the 40 acylcarnitines. Results are reported as mean, standard deviation, 25th and 75th percentile (inter-quartile range), minimum and maximum (extreme values). For each compound the limit of detection is reported along with the number of subjects with quantifiable concentrations.

	**Mean±SD (µmol/L)**	**Median**	**Inter-quartile Range**	**Extreme values**	**LOD**	**N<LOD (%)**
L-Carnitine (C0)	32.66±7.26	32.15	[27.70;37.10]	(12.00;73.20)	1.59	0 (0%)
Acetylcarnitine (C2)	5.83±1.96	5.48	[4.43;6.86]	(1.87;19.80)	0.11	0 (0%)
Propionylcarnitine (C3)	0.36±0.12	0.34	[0.28;0.42]	(<LOD;1.30)	0.05	7 (0.5%)
Hydroxybutyrylcarnitine (C3-DC (C4-OH))	<LOD	<LOD	<LOD	(<LOD;0.30)	0.04	1163 (83.5%)
Hydroxypropionylcarnitine (C3-OH)	0.26±0.16	<LOD	<LOD	(<LOD;2.76)	0.25	1306 (93.8%)
Propenoylcarnitine (C3:1)	<LOD	<LOD	<LOD	(<LOD;0.60)	0.07	1323 (95%)
Butyrylcarnitine (C4)	0.20±0.13	0.18	[<LOD;0.25]	(<LOD;1.33)	0.10	480 (34.5%)
Butenylcarnitine (C4:1)	<LOD	<LOD	<LOD	(<LOD;1.29)	0.06	1333 (95.7%)
Valerylcarnitine (C5)	0.16±0.11	<LOD	[<LOD;0.17]	(<LOD;3.86)	0.14	978 (70.1%)
Glutarylcarnitine (Hydroxyhexanoylcarnitine) (C5-DC (C6-OH))	<LOD	<LOD	<LOD	(<LOD;0.13)	0.05	1269 (91.1%)
Methylglutarylcarnitine (C5-M-DC)	<LOD	<LOD	<LOD	(<LOD;0.14)	0.05	1275 (91.5%)
Hydroxyvalerylcarnitine (Methylmalonylcarnitine) (C5-OH (C3-DC-M))	<LOD	<LOD	<LOD	(<LOD;0.23)	0.09	1283 (92.1%)
Tiglylcarnitine (C5:1)	0.08±0.12	<LOD	<LOD	(<LOD;1.54)	0.07	1293 (92.8%)
Glutaconylcarnitine (C5:1-DC)	<LOD	<LOD	<LOD	(<LOD;0.10)	0.05	1326 (95.2%)
Hexanoylcarnitine (Fumarylcarnitine) (C6 (C4:1-DC))	0.08±0.03	<LOD	<LOD	(<LOD;0.84)	0.07	1108 (79.5%)
Hexenoylcarnitine (C6:1)	<LOD	<LOD	<LOD	(<LOD;0.13)	0.05	1334 (95.8%)

Pimelylcarnitine (C7-DC)	0.04±0.01	<LOD	<LOD	(<LOD;0.16)	0.03	1156 (83%)
Octanoylcarnitine (C8)	0.19±0.11	<LOD	[<LOD;0.22]	(<LOD;2.23)	0.14	902 (64.8%)
Nonaylcarnitine (C9)	<LOD	<LOD	<LOD	(<LOD;0.38)	0.06	1374 (98.6%)
Decanoylcarnitine (C10)	0.53±0.12	<LOD	<LOD	(<LOD;2.85)	0.51	1349 (96.8%)
Decenoylcarnitine (C10:1)	0.12±0.04	<LOD	<LOD	(<LOD;0.45)	0.11	1154 (82.8%)
Decadienylcarnitine (C10:2)	<LOD	<LOD	<LOD	(<LOD;0.21)	0.09	1384 (99.4%)
Dodecanoylcarnitine (C12)	0.10±0.04	<LOD	<LOD	(<LOD;0.45)	0.08	1071 (76.9%)
Dodecanedioylcarnitine (C12-DC)	<LOD	<LOD	<LOD	<LOD	0.66	1384 (99.4%)
Dodecenoylcarnitine (C12:1)	0.14±0.08	0.11	[<LOD;0.18]	(<LOD;0.73)	0.08	638 (45.8%)
Tetradecanoylcarnitine (C14)	<LOD	<LOD	<LOD	(<LOD;0.32)	0.08	1389 (99.7%)
Hydroxytetradecenoylcarnitine (C14:1-OH)	<LOD	<LOD	<LOD	(<LOD;0.10)	0.03	1371 (98.4%)
Tetradecenoylcarnitine (C14:1)	0.09±0.07	0.08	[0.05;0.12]	(<LOD;1.12)	0.02	316 (22.7%)
Tetradecadienylcarnitine (C14:2)	<LOD	<LOD	<LOD	(<LOD;0.21)	0.03	1162 (83.4%)
Hydroxytetradecadienylcarnitine (C14:2-OH)	<LOD	<LOD	<LOD	(<LOD;0.14)	0.03	1338 (96.1%)
Hexadecanoylcarnitine (C16)	0.11±0.06	0.11	[<LOD;0.15]	(<LOD;0.53)	0.05	591 (42.4%)
Hydroxyhexadecanoylcarnitine (C16-OH)	<LOD	<LOD	<LOD	(<LOD;0.16)	0.07	1388 (99.6%)
Hydroxyhexadecenoylcarnitine (C16:1-OH)	<LOD	<LOD	<LOD	(<LOD;1.45)	0.03	1374 (98.6%)
Hexadecenoylcarnitine (C16:1)	<LOD	<LOD	<LOD	(<LOD;0.22)	0.04	1298 (93.2%)
Hexadecadienylcarnitine (C16:2)	<LOD	<LOD	<LOD	(<LOD;0.07)	0.03	1304 (93.6%)
Hydroxyhexadecadienylcarnitine (C16:2-OH)	<LOD	<LOD	<LOD	(<LOD;0.09)	0.04	1330 (95.5%)
Octadecanoylcarnitine (C18)	<LOD	<LOD	<LOD	(<LOD;0.28)	0.04	1273 (91.4%)
Octadecenoylcarnitine (C18:1)	0.15±0.04	0.14	[0.12;0.17]	(<LOD;0.30)	0.04	12 (0.9%)

Hydroxyoctadecenoylcarnitine (C18:1-OH)	<LOD	<LOD	<LOD	(<LOD;0.09)	0.09	1393 (100%)
Octadecadienylcarnitine (C18:2)	0.05±0.02	0.04	[<LOD;0.06]	(<LOD;0.20)	0.03	647 (46.4%)

SD = standard deviation, LOD = limit of detection

Table S5 - Results obtained from the analyses of the 14 lysophosphatidylcholine. Results are reported as mean, standard deviation, 25th and 75th percentile (inter-quartile range), minimum and maximum (extreme values). For each compound the limit of detection is reported along with the number of subjects with quantifiable concentrations.

	**Mean±SD (µmol/L)**	**Median**	**Inter-quartile Range**	**Extreme values**	**LOD**	**N<LOD (%)**
lysoPC a C14:0	<LOD	<LOD	<LOD	<LOD	12.10	1393 (100%)
lysoPC a C16:0	85.84±21.44	83.10	[70.88;97.40]	(39.20;211.00)	0.43	0 (0%)
lysoPC a C16:1	2.63±0.86	2.51	[2.02;3.02]	(0.93;7.80)	0.07	0 (0%)
lysoPC a C17:0	1.51±0.45	1.46	[1.17;1.76]	(0.50;3.31)	0.05	0 (0%)
lysoPC a C18:0	26.74±7.64	25.80	[21.50;30.90]	(6.46;62.00)	0.27	0 (0%)
lysoPC a C18:1	16.76±4.84	16.10	[13.30;19.30]	(5.47;45.60)	0.16	0 (0%)
lysoPC a C18:2	18.51±6.03	17.45	[14.40;21.80]	(6.33;48.30)	0.16	0 (0%)
lysoPC a C20:3	2.12±0.62	2.05	[1.69;2.46]	(0.88;5.21)	0.15	0 (0%)
lysoPC a C20:4	5.38±1.65	5.18	[4.21;6.28]	(1.47;12.80)	0.05	0 (0%)
lysoPC a C24:0	0.23±0.14	0.24	[0.15;0.32]	(<LOD;0.80)	0.03	327 (23.5%)
lysoPC a C26:0	0.13±0.06	0.11	[0.09; 0.16]	(<LOD;0.46)	0.03	85 (6.1%)
lysoPC a C26:1	0.08±0.06	0.07	[<LOD; 0.12]	(<LOD;0.38)	0.02	590 (42.4%)
lysoPC a C28:0	0.16±0.12	0.14	[0.10;0.18]	(<LOD;1.29)	0.06	151 (10.8%)
lysoPC a C28:1	0.22±0.15	0.18	[0.12;0.32]	(<LOD;0.90)	0.03	198 (14.2%)

SD = standard deviation, LOD = limit of detection

Table S6 - Results obtained from the analyses of the 76 phosphatidylcholines. Results are reported as mean, standard deviation, 25th and 75th percentile (inter-quartile range), minimum and maximum (extreme values). For each compound the limit of detection is reported along with the number of subjects with quantifiable concentrations.

	Mean±SD (µmol/L)	Median	Inter-quartile Range	Extreme values	LOD	N<LOD (%)
PC aa C24:0	0.17±0.10	0.14	[0.10;0.21]	(<LOD;0.99)	0.07	216 (15.5%)
PC aa C26:0	0.53±0.43	0.42	[<LOD; 0.70]	(<LOD;3.26)	0.80	509 (36.5%)
PC aa C28:1	2.76±0.86	2.69	[2.22;3.24]	(<LOD;6.49)	0.06	29 (2.1%)
PC aa C30:0	3.68±1.31	3.51	[2.73;4.44]	(0.91;10.90)	0.07	0 (0%)
PC aa C30:2	0.04±0.06	<LOD	[<LOD;0.06]	(<LOD;0.77)	0.01	865 (62.1%)
PC aa C32:0	12.10±3.10	11.80	[10.00;14.00]	(4.28;25.60)	0.06	0 (0%)
PC aa C32:1	13.95±7.19	12.60	[8.94;16.93]	(1.81;61.30)	0.03	0 (0%)
PC aa C32:2	3.47±1.45	3.28	[2.44;4.16]	(0.59;10.50)	0.01	0 (0%)
PC aa C32:3	0.34±0.09	0.32	[0.27;0.39]	(0.13;0.74)	0.01	0 (0%)
PC aa C34:1	200.32±53.12	196.00	[163.00;232.25]	(70.70;480.00)	0.20	0 (0%)
PC aa C34:2	274.97±58.21	273.50	[235.00;310.00]	(90.80;600.00)	0.21	0 (0%)
PC aa C34:3	10.86±3.35	10.50	[8.44;12.70]	(3.44;35.30)	0.13	0 (0%)
PC aa C34:4	1.34±0.49	1.28	[0.99;1.61]	(0.28;4.18)	0.01	0 (0%)
PC aa C36:0	1.80±0.78	1.64	[1.25;2.20]	(<LOD;6.35)	0.31	8 (0.6%)
PC aa C36:1	52.91±16.64	50.65	[41.20;62.50]	(17.10;135.00)	0.09	0 (0%)
PC aa C36:2	200.41±44.99	198.00	[168.00;230.00]	(75.00;377.00)	0.11	0 (0%)
PC aa C36:3	124.52±31.90	122.00	[103.00;142.00]	(46.10;272.00)	0.07	0 (0%)
PC aa C36:4	157.57±42.23	153.00	[127.00;182.00]	(64.90;450.00)	0.07	0 (0%)
PC aa C36:5	16.89±10.13	14.50	[10.60;20.30]	(3.35;107.00)	0.03	0 (0%)
PC aa C36:6	0.72±0.29	0.67	[0.50;0.86]	(0.16;2.75)	0.02	0 (0%)
PC aa C38:0	2.36±0.76	2.22	[1.82;2.78]	(0.76;6.96)	0.04	0 (0%)
PC aa C38:1	0.97±1.40	0.61	[0.32;1.06]	(<LOD;24.90)	0.01	150 (10.8%)

PC aa C38:3	52.23±15.21	51.30	[41.10;61.23]	(15.00;135.00)	0.06	0 (0%)
PC aa C38:4	96.50±26.26	93.65	[77.48;113.00]	(35.90;237.00)	0.05	0 (0%)
PC aa C38:5	39.94±11.27	38.40	[31.78;46.63]	(16.40,94.80)	0.04	0 (0%)
PC aa C38:6	59.65±20.59	56.25	[45.20;70.83]	(18.00;172.00)	0.04	0 (0%)
PC aa C40:1	0.19±0.10	<LOD	<LOD	(<LOD;0.99)	0.15	1104 (79.3%)
PC aa C40:2	0.21±0.14	0.19	[0.16;0.23]	(<LOD;2.09)	0.02	2 (0.1%)
PC aa C40:3	0.38±0.14	0.35	[0.30;0.42]	(0.15;1.75)	0.01	0 (0%)
PC aa C40:4	2.85±0.93	2.71	[2.19;3.38]	(0.85;8.22)	0.02	0 (0%)
PC aa C40:5	7.68±2.42	7.30	[5.94;9.16]	(2.43;18.80)	0.01	0 (0%)
PC aa C40:6	23.10±8.28	21.55	[17.40;27.83]	(7.39;77.90)	0.25	0 (0%)
PC aa C42:0	0.35±0.09	0.34	[0.29;0.40]	(<LOD;0.74)	0.05	2 (0.1%)
PC aa C42:1	0.13±0.04	0.13	[0.11;0.15]	(<LOD;0.32)	0.01	6 (0.4%)
PC aa C42:2	0.16±0.06	0.16	[0.14;0.20]	(<LOD;0.41)	0.03	149 (10.7%)
PC aa C42:4	0.13±0.04	0.12	[0.10;0.14]	(<LOD;0.46)	0.02	9 (0.6%)
PC aa C42:5	0.18±0.05	0.18	[0.15;0.21]	(<LOD;0.63)	0.003	2 (0.1%)
PC aa C42:6	0.27±0.08	0.26	[0.21;0.32]	(<LOD;0.73)	0.17	158 (11.3%)
PC ae C30:0	0.28±0.09	0.27	[0.22;0.33]	(<LOD;0.74)	0.05	3 (0.2%)
PC ae C30:1	0.09±0.07	0.08	[0.04;0.12]	(<LOD;0.50)	0.01	223 (16%)
PC ae C30:2	0.10±0.03	0.09	[0.08;0.11]	(<LOD;0.24)	0.01	5 (0.4%)
PC ae C32:1	2.39±0.61	2.33	[1.96;2.77]	(0.87;4.83)	0.01	0 (0%)
PC ae C32:2	0.65±0.18	0.62	[0.53;0.75]	(0.25;1.53)	0.06	0 (0%)
PC ae C34:0	1.17±0.35	1.12	[0.92;1.36]	(0.35;2.76)	0.03	0 (0%)
PC ae C34:1	8.31±2.04	8.10	[6.87;9.58]	(2.96;18.60)	0.02	0 (0%)
PC ae C34:2	9.15±2.62	8.85	[7.30;10.70]	(2.95;22.50)	0.02	0 (0%)
PC ae C34:3	5.90±1.93	5.69	[4.52;6.98]	(1.99;14.90)	0.04	0 (0%)
PC ae C36:0	0.63±0.16	0.62	[0.52;0.73]	(<LOD;1.32)	0.13	1 (0.1%)

PC ae C36:1	5.60±1.52	5.37	[4.56;6.42]	(2.07;16.50)	0.06	0 (0%)
PC ae C36:2	10.30±2.64	9.98	[8.39;11.90]	(4.15;20.80)	0.02	0 (0%)
PC ae C36:3	6.10±1.66	5.94	[4.92;7.06]	(2.29;13.60)	0.01	0 (0%)
PC ae C36:4	16.61±4.45	16.20	[13.50;19.10]	(6.77;42.90)	0.14	0 (0%)
PC ae C36:5	10.15±3.01	9.71	[8.02;11.80]	(3.90;26.00)	0.02	0 (0%)
PC ae C38:0	1.90±0.66	1.78	[1.46;2.25]	(0.58;5.90)	0.08	0 (0%)
PC ae C38:1	0.31±0.36	0.26	[0.11;0.42]	(<LOD;4.35)	0.003	227 (16.3%)
PC ae C38:2	1.34±0.56	1.21	[1.01;1.53]	(0.50;6.60)	0.02	0 (0%)
PC ae C38:3	2.91±1.00	2.74	[2.29;3.31]	(0.97;12.40)	0.02	0 (0%)
PC ae C38:4	10.90±2.54	10.70	[9.03;12.40]	(4.05;21.80)	0.14	0 (0%)
PC ae C38:5	13.76±3.26	13.30	[11.40;15.90]	(6.65;26.00)	0.03	0 (0%)
PC ae C38:6	6.16±1.82	5.91	[4.88;7.15]	(2.36;16.90)	0.01	0 (0%)
PC ae C40:1	0.95±0.27	0.92	[0.75;1.11]	(0.31;2.06)	0.01	0 (0%)
PC ae C40:2	1.21±0.35	1.16	[0.98;1.39]	(0.43;3.57)	0.03	0 (0%)
PC ae C40:3	0.72±0.35	0.65	[0.55;0.76]	(0.26;4.08)	0.02	0 (0%)
PC ae C40:4	1.52±0.41	1.47	[1.26;1.68]	(0.54;4.87)	0.09	0 (0%)
PC ae C40:5	2.14±0.57	2.05	[1.77;2.40]	(0.99;6.63)	0.02	0 (0%)
PC ae C40:6	3.27±0.96	3.12	[2.58;3.82]	(1.29;8.08)	0.02	0 (0%)
PC ae C42:0	<LOD	<LOD	<LOD	(<LOD;1.38)	0.67	1380 (99.1%)
PC ae C42:1	0.24±0.07	0.23	[0.19;0.27]	(<LOD;0.84)	0.07	26 (1.9%)
PC ae C42:2	0.37±0.10	0.36	[0.30;0.43]	(0.13;1.01)	0.02	0 (0%)
PC ae C42:3	0.49±0.13	0.47	[0.41;0.55]	(0.22;1.19)	0.03	0 (0%)
PC ae C42:4	0.55±0.13	0.53	[0.46;0.61]	(0.24;1.28)	0.02	0 (0%)
PC ae C42:5	1.38±0.32	1.34	[1.16;1.54]	(<LOD;3.33)	0.64	4 (0.3%)
PC ae C44:3	0.05±0.04	0.04	[<LOD;0.06]	(<LOD;0.38)	0.08	626 (44.9%)
PC ae C44:4	0.21±0.05	0.20	[0.17;0.24]	(<LOD;0.54)	0.04	2 (0.1%)

PC ae C44:5	1.22±0.32	1.18	[0.99;1.40]	(<LOD;3.91)	0.03	1 (0.1%)
PC ae C44:6	0.85±0.21	0.82	[0.70;0.97]	(0.36;2.23)	0.08	0 (0%)

aa: Diacylphosphatidylcholine – ae: Acylalkylphosphatidylcholine

SD = standard deviation, LOD = limit of detection

Table S7 - Results obtained from the analyses of the 15 sphingomyelin. Results are reported as mean, standard deviation, 25th and 75th percentile (inter-quartile range), minimum and maximum (extreme values). For each compound the limit of detection is reported along with the number of subjects with quantifiable concentrations.

	**Mean±SD (µmol/L)**	**Median**	**Inter-quartile Range**	**Extreme values**	**LOD**	**N<LOD (%)**
SM (OH) C14:1	6.89±1.85	6.74	[5.59;7.90]	(2.52;16.30)	0.02	0 (0%)
SM (OH) C16:1	3.76±1.04	3.61	[3.03;4.36]	(1.35;8.60)	0.004	0 (0%)
SM (OH) C22:1	14.68±3.99	14.30	[11.90;17.00]	(5.86;37.70)	0.05	0 (0%)
SM (OH) C22:2	13.24±3.50	13.00	[10.80;15.50]	(4.61;28.40)	0.01	0 (0%)
SM (OH) C24:1	1.22±0.34	1.18	[0.98;1.43]	(<LOD;2.56)	0.01	1 (0.1%)
SM C16:0	122.45±27.33	122.00	[104.00;138.00]	(49.70;237.00)	0.04	0 (0%)
SM C16:1	19.14±4.60	18.80	[16.00;21.90]	(8.08;39.50)	0.02	0 (0%)
SM C18:0	29.34±7.58	28.40	[23.90;33.90]	(10.20;68.20)	0.01	0 (0%)
SM C18:1	14.58±3.97	14.25	[11.80;16.90]	(5.66;36.30)	0.004	0 (0%)
SM C20:2	0.42±0.14	0.40	[0.32;0.49]	(0.13;1.02)	0.01	0 (0%)
SM C22:3	0.13±0.12	0.12	[0.01;0.19]	(<LOD;0.94)	0.004	339 (24.3%)
SM C24:0	21.63±5.46	21.15	[17.60;24.80]	(8.57;47.30)	0.07	0 (0%)
SM C24:1	59.06±15.34	57.35	[48.88;68.20]	(21.60;1380.00)	0.03	0 (0%)
SM C26:0	0.09±0.04	0.09	[0.07;0.12]	(<LOD;0.30)	0.003	107 (7.7%)
SM C26:1	0.22±0.08	0.21	[0.17;0.27]	(0.06;0.66)	0.01	0 (0%)

SD = standard deviation, LOD = limit of detection
