# Supplementary figures and images for: Plasma Metabolomic Profiling in 1391 Subjects with Overweight and Obesity from the SPHERE Study
Source: Metabolites. 2021 Mar 24;11(4):194. doi: 10.3390/metabo11040194 (PMC8064361; doi:10.3390/metabo11040194)

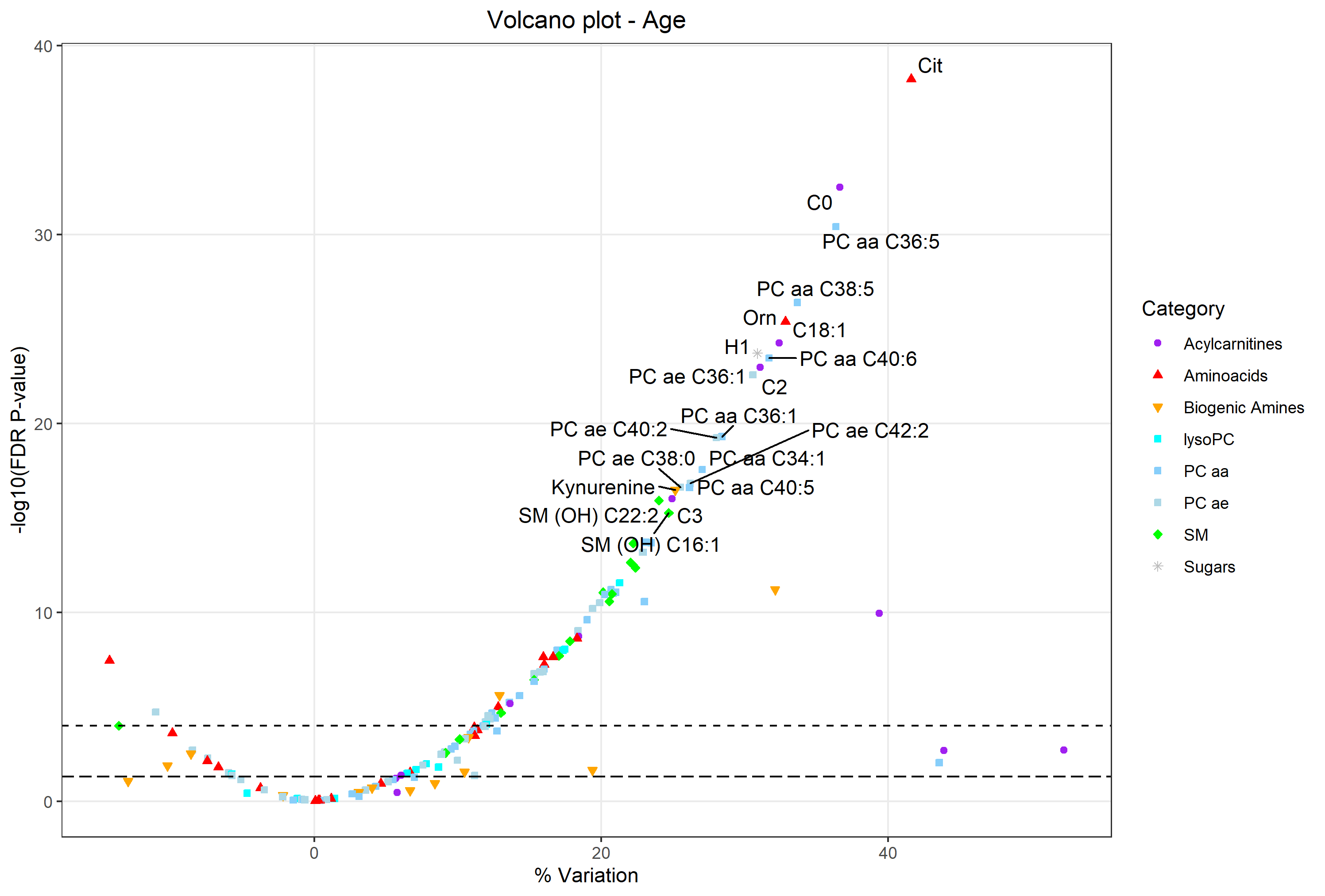

Supplement: Supplementary file 1 [file metabolites-11-00194-s001.zip › Fig_S01.png]

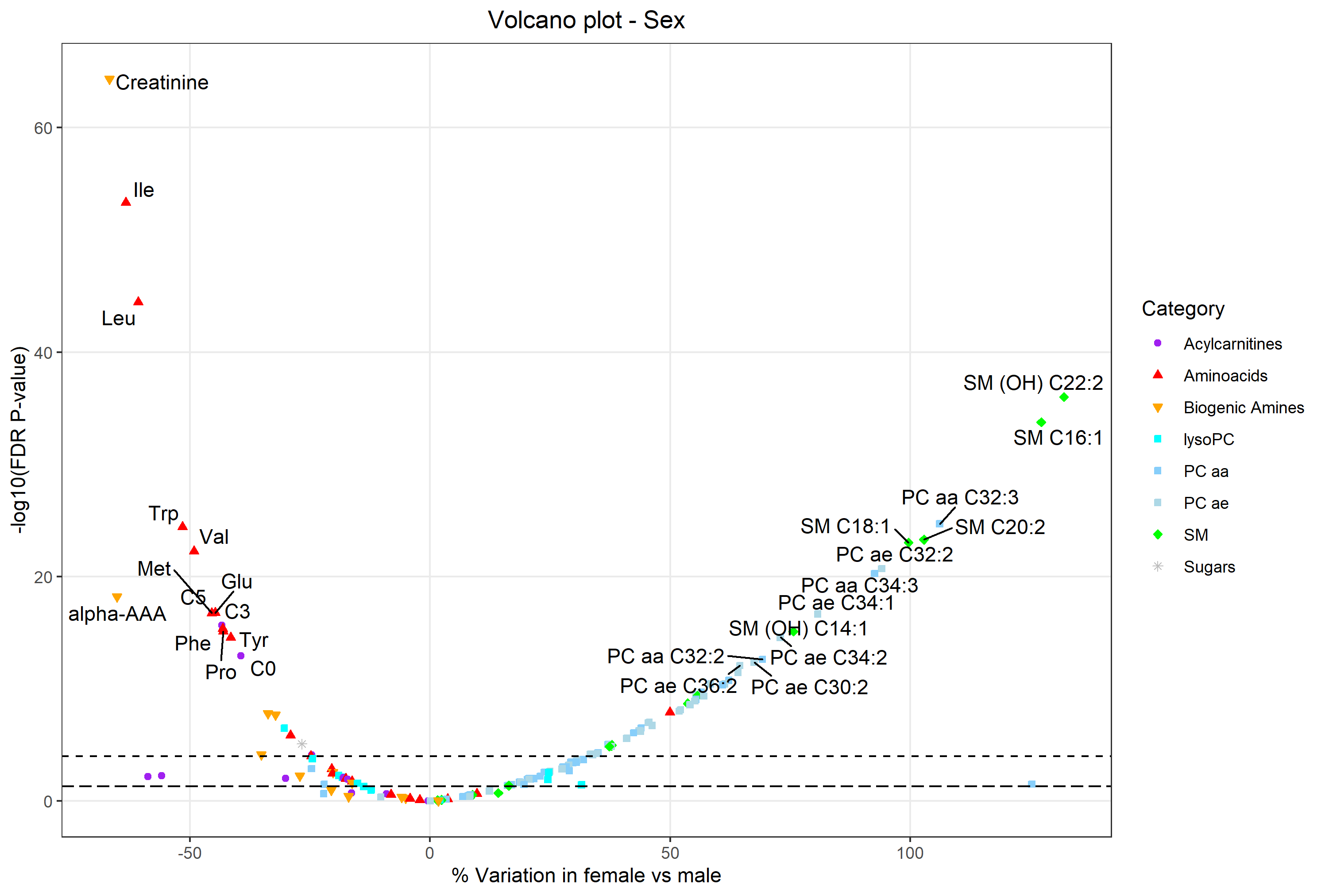

Supplement: Supplementary file 1 [file metabolites-11-00194-s001.zip › Fig_S02.png]

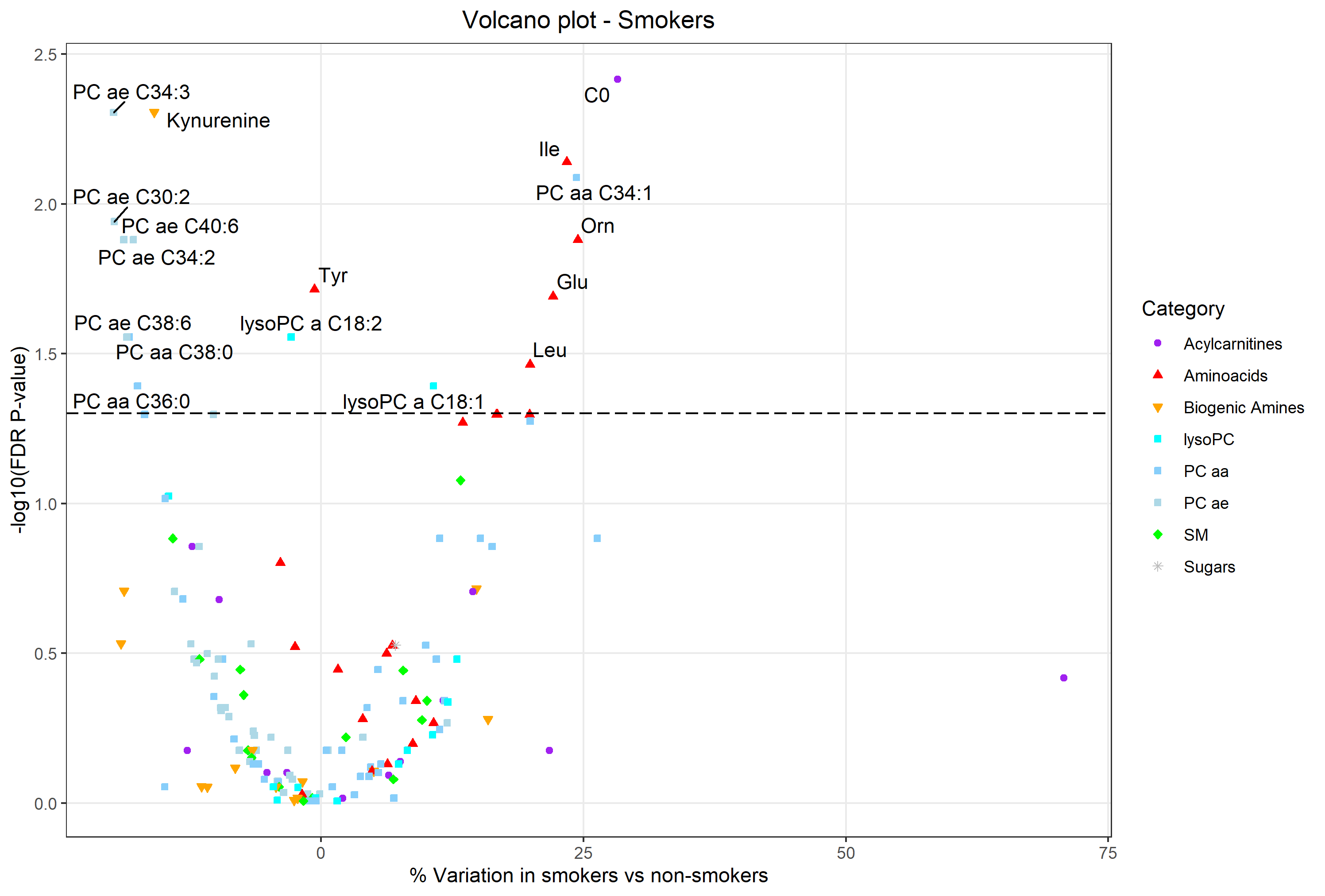

Supplement: Supplementary file 1 [file metabolites-11-00194-s001.zip › Fig_S03.png]

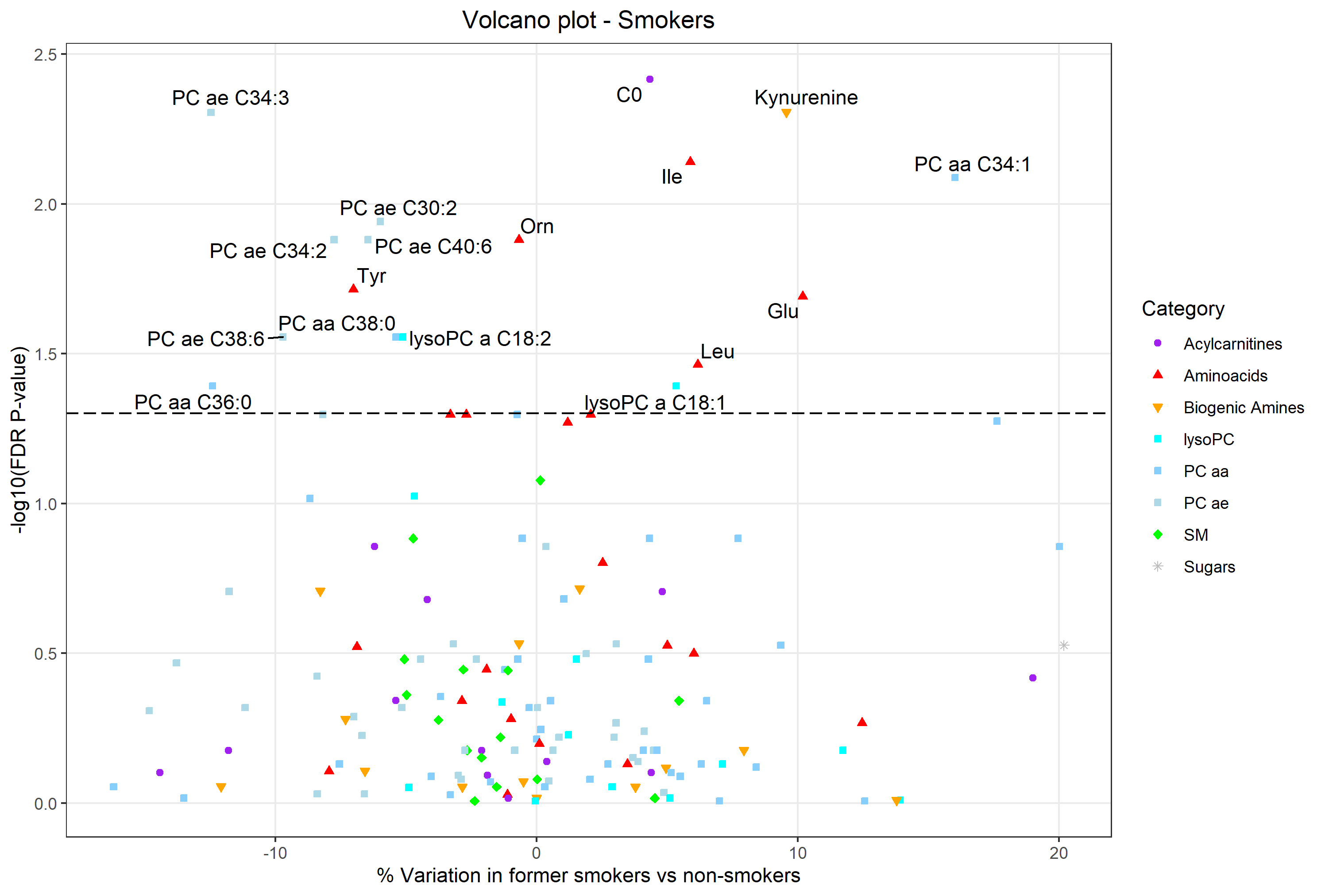

Supplement: Supplementary file 1 [file metabolites-11-00194-s001.zip › Fig_S04.png]

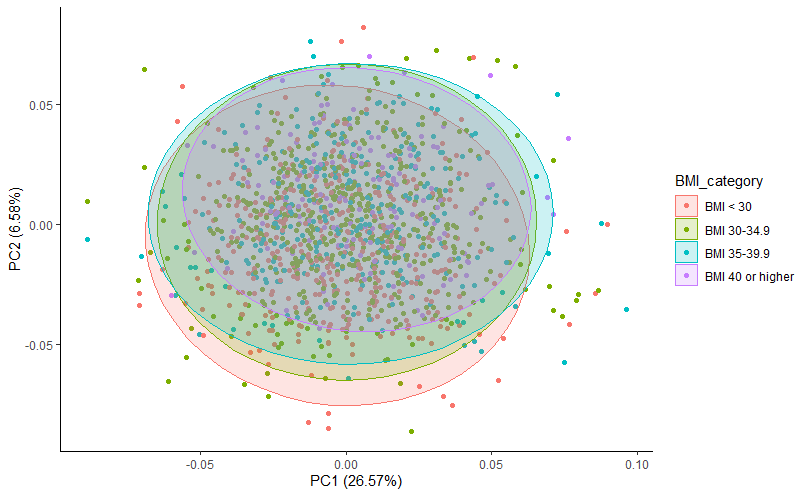

Supplement: Supplementary file 1 [file metabolites-11-00194-s001.zip › Fig_S05.png]

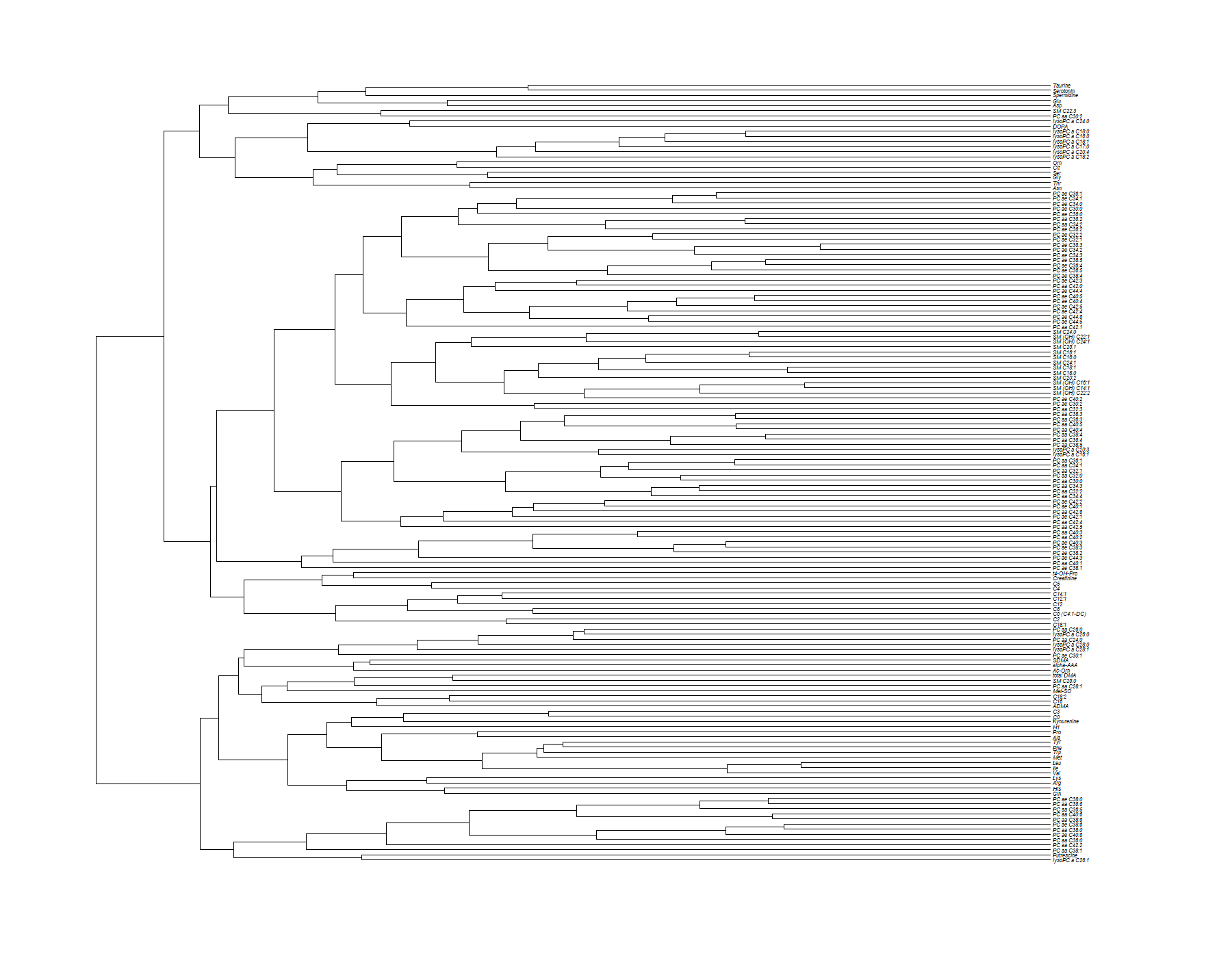

Supplement: Supplementary file 1 [file metabolites-11-00194-s001.zip › Fig_S06.png]

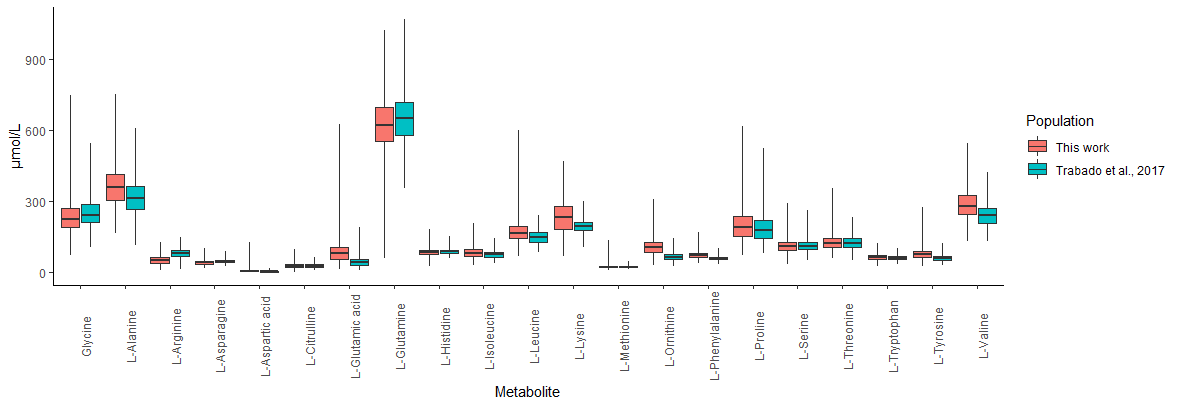

Supplement: Supplementary file 1 [file metabolites-11-00194-s001.zip › FigS07_aa.png]

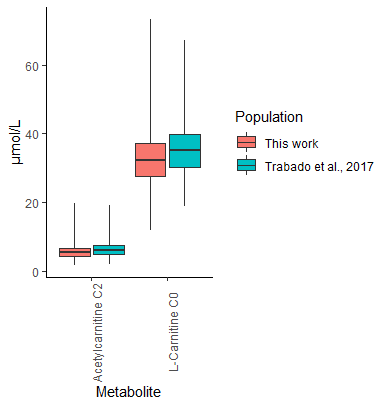

Supplement: Supplementary file 1 [file metabolites-11-00194-s001.zip › FigS08_AC1.png]

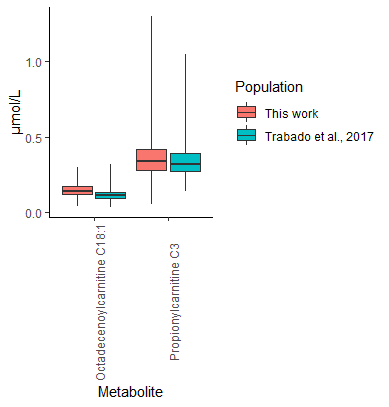

Supplement: Supplementary file 1 [file metabolites-11-00194-s001.zip › FigS09_AC2.png]

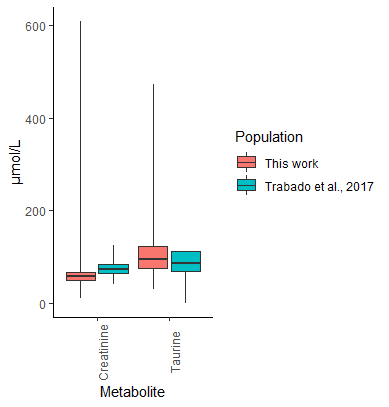

Supplement: Supplementary file 1 [file metabolites-11-00194-s001.zip › FigS10_BA1.png]

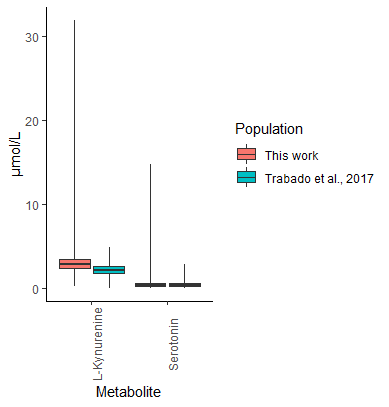

Supplement: Supplementary file 1 [file metabolites-11-00194-s001.zip › FigS11_BA2.png]

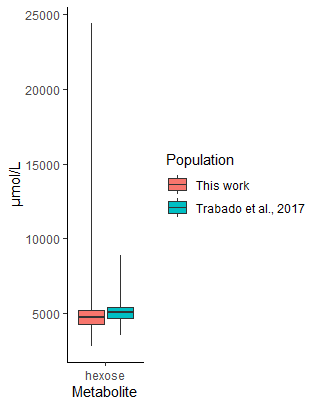

Supplement: Supplementary file 1 [file metabolites-11-00194-s001.zip › FigS12_hexose.png]

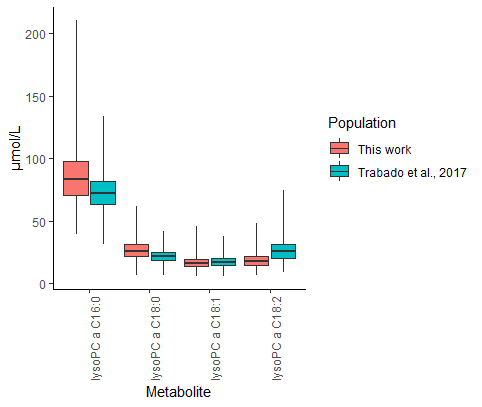

Supplement: Supplementary file 1 [file metabolites-11-00194-s001.zip › FigS13_LPC1.png]

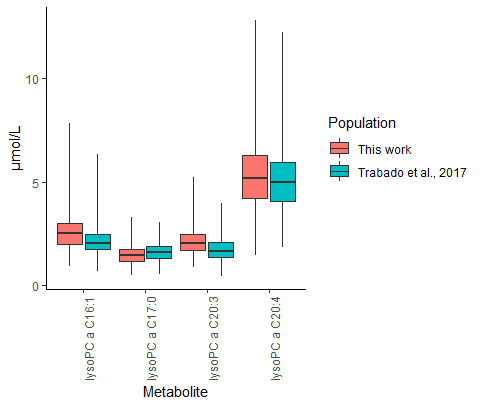

Supplement: Supplementary file 1 [file metabolites-11-00194-s001.zip › FigS14_LPC2.png]

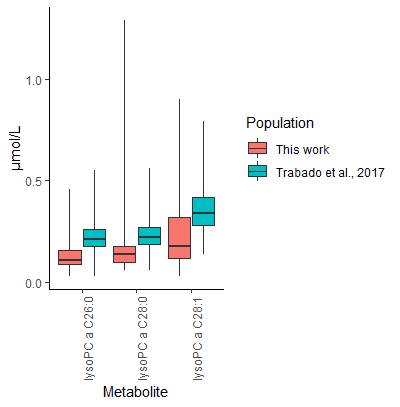

Supplement: Supplementary file 1 [file metabolites-11-00194-s001.zip › FigS15_LPC3.png]

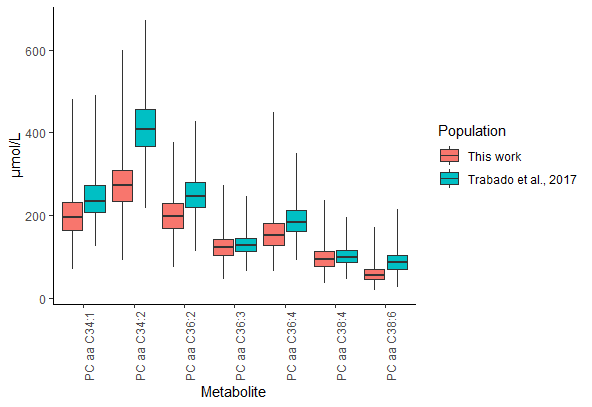

Supplement: Supplementary file 1 [file metabolites-11-00194-s001.zip › FigS16_PCaa1.png]

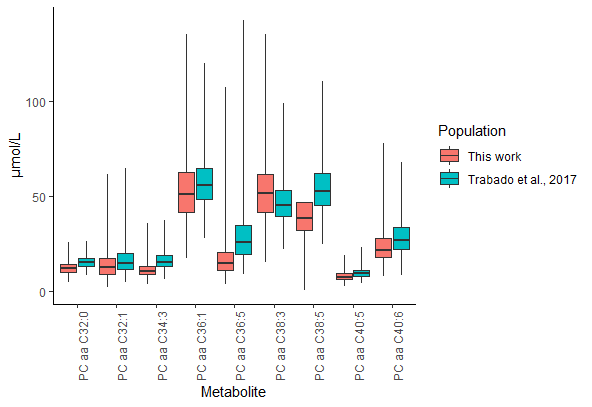

Supplement: Supplementary file 1 [file metabolites-11-00194-s001.zip › FigS17_PCaa2.png]

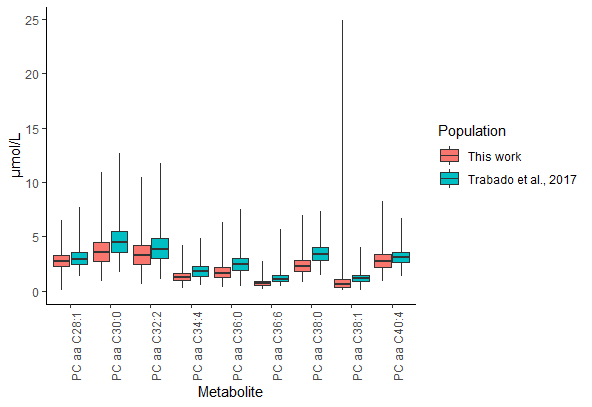

Supplement: Supplementary file 1 [file metabolites-11-00194-s001.zip › FigS18_PCaa3.png]

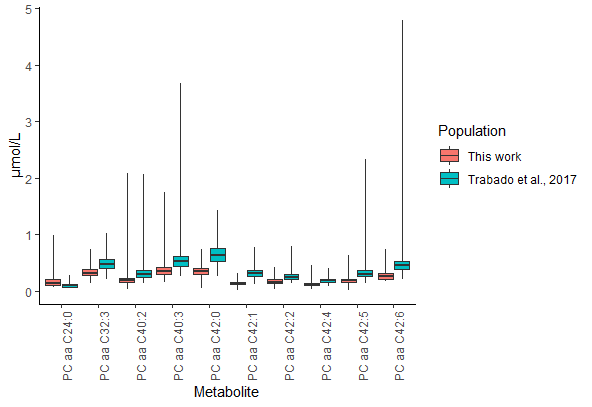

Supplement: Supplementary file 1 [file metabolites-11-00194-s001.zip › FigS19_PCaa4.png]

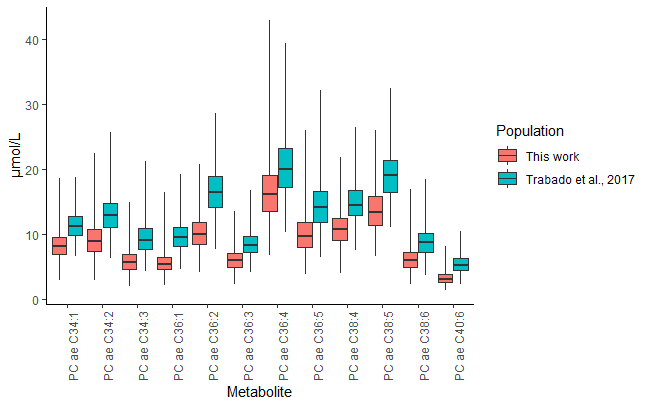

Supplement: Supplementary file 1 [file metabolites-11-00194-s001.zip › FigS20_PCae1.png]

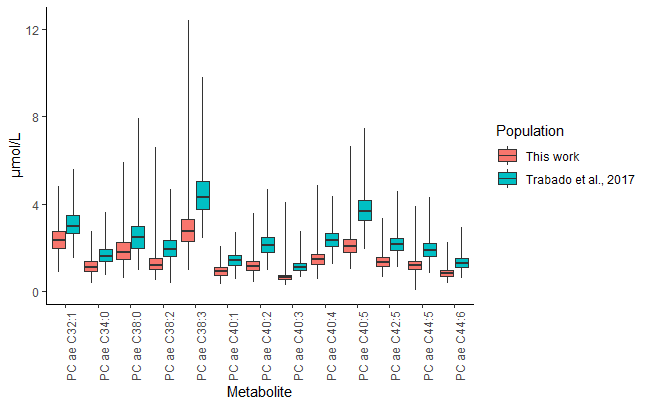

Supplement: Supplementary file 1 [file metabolites-11-00194-s001.zip › FigS21_PCae2.png]

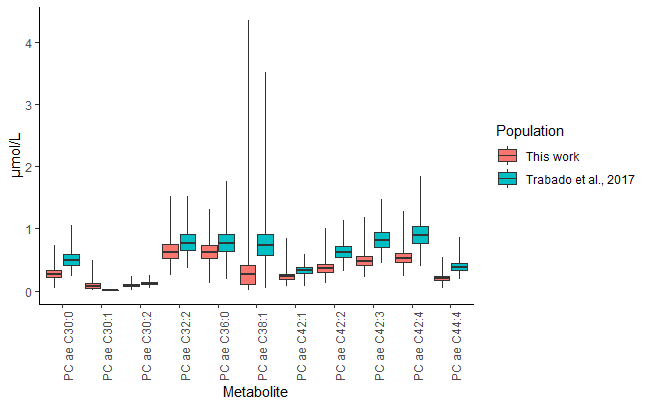

Supplement: Supplementary file 1 [file metabolites-11-00194-s001.zip › FigS22_PCae3.png]

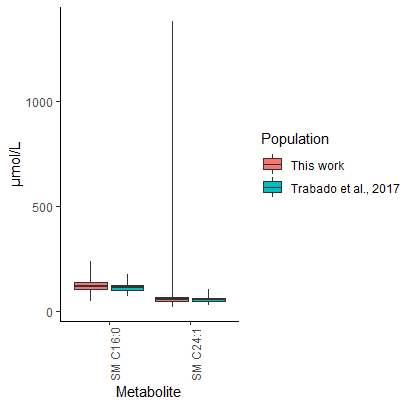

Supplement: Supplementary file 1 [file metabolites-11-00194-s001.zip › FigS23_SM1.png]

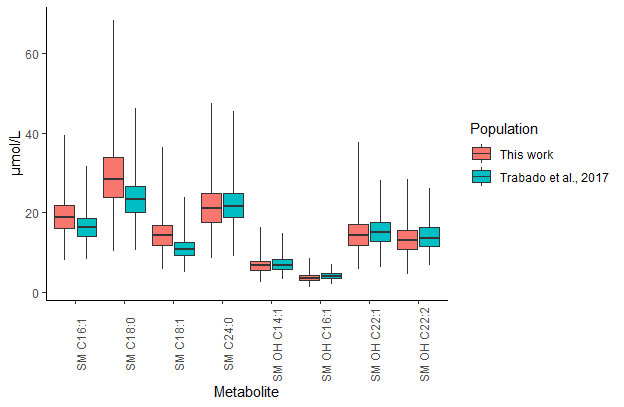

Supplement: Supplementary file 1 [file metabolites-11-00194-s001.zip › FigS24_SM2.png]

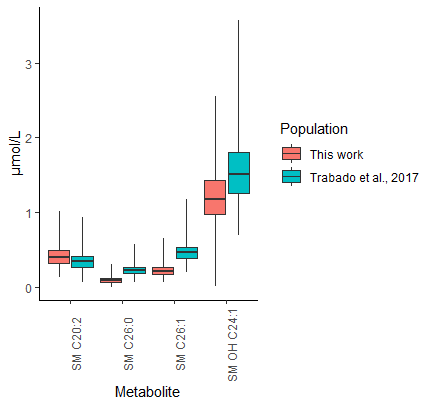

Supplement: Supplementary file 1 [file metabolites-11-00194-s001.zip › FigS25_SM3.png]
